# TAFRO Syndrome—A Decade Later, Still Racing Against Time

**DOI:** 10.3390/biomedicines13092303

**Published:** 2025-09-19

**Authors:** Yasufumi Masaki

**Affiliations:** Department of Hematology and Immunology, Medicine, Kanazawa Medical University, 1-1 Daigaku, Uchinada, Kahoku-gun, Ishikawa 920-0293, Japan; yasum@kanazawa-med.ac.jp; Tel.: +81-76-218-8158; Fax: +81-76-286-9290

Since its first description in Japan in 2010 [1], TAFRO (thrombocytopenia, anasarca, fever, reticulin fibrosis/renal insufficiency, and organomegaly) syndrome, driven by an acute hypercytokinemic storm, has emerged as a formidable clinical challenge. Its course can be precipitous and its outcome unforgiving. Its phenotype overlaps with malignant, autoimmune, and infectious disorders, demanding diagnostic vigilance from the outset [2].

Pathologically, lymph nodes may show Castleman disease-like changes, but many patients present without accessible or conspicuous nodal disease [3]. The consensus has become increasingly clear: TAFRO syndrome is not a histologic variant of Castleman disease, but a clinically distinct entity with its own trajectory and prognosis [4].

The pathophysiology of TAFRO syndrome is complex. IL-6 and VEGF are central early drivers, but multiple signaling axes—JAK–STAT, PI3K/AKT/mTOR, NF-κB—appear to intersect [5], while other factors, such as IL-1β, TNF-α, IL-10, IL-23, CXCL10(IP–10), CXCL13, SAA, NPS-PLA2, and IGFBP-1, may underpin the syndrome’s acute severity. Severity classifications [6] and prognostic models such as the TS-PSS (TAFRO Syndrome Prognostic Scoring System) [7] provide a framework for risk stratification; however, improving survival, particularly in severe cases, remains a challenge.

A universally accepted therapeutic standard has not been established. High-dose glucocorticoids are the first-line treatment but are often insufficient. Biologic agents, calcineurin inhibitors, cytotoxic regimens, and targeted pathway inhibitors have shown promise in selected patients. The reports in this Special Issue underscore both the ingenuity of clinicians and the pressing need for prospective, collaborative trials.

TAFRO syndrome demands urgency—not only in diagnosis and initiation of therapy, but also in research to identify disease-specific biomarkers and truly disease-modifying treatments. More than a decade since its recognition, the task before us is clear: to move from reactive, case-by-case management to a strategic, evidence-based framework capable of altering the natural course of this devastating condition.
